# The differences in whole-body sagittal alignment between different postures in young, healthy adults

**DOI:** 10.1186/s12891-020-03715-2

**Published:** 2020-10-20

**Authors:** Rui Xue, Dai Liu, Yong Shen

**Affiliations:** 1 Department of Spine Surgery, The Third Hospital of HeBei Medical University, 139 Ziqiang Road, Shi Jiazhuang, 050051 China; 2 Rehabilitation Office, The Third Hospital of HeBei Medical University, 139 Ziqiang Road, Shi Jiazhuang, 050051 China

**Keywords:** Standard upright position, Natural and comfortable upright position, Spinal profile, Cranial center vertical axis

## Abstract

**Study design:**

Prospective study.

**Objective:**

To identify the radiographic differences between the standard upright position and the natural and comfortable upright position.

**Methods:**

The radiographic data of 50 young and healthy adults were evaluated, and parameters including the global cervical angle (GCA), global thoracic angle (GTA), global lumbar angle (GLA) were used to depict the spine profile; the distance from the cranial center to the posterior corner of S1 (CSVA-S), the center of the hip (CSVA-H), the center of the knee (CSVA-K) and the center of the ankle (CSVA-A) were measured in both the standard and the natural and comfortable upright positions to assess whole-body balance.

**Results:**

Significant differences were observed in the GCA (17.39 ± 6.90 vs. 10.90 ± 3.77, *p* < .001), GTA (25.63 ± 7.27 vs. 45.42 ± 8.15 *p* < .001), GLA (42.64 ± 8.05 vs. 20.21 ± 7.47 *p* < .001), CSVA-S (0.33 ± 2.76 cm vs. 8.54 ± 3.78 cm, *p* < 0.001), CSVA-H (1.53 ± 3.11 cm vs. 5.71 ± 3.26 cm, *p* < 0.001), CSVA-K (3.58 ± 2.47 cm vs. 5.22 ± 2.69 cm, *p* = 0.002) and CSVA-A (1.79 ± 1.92 cm vs. 4.79 ± 2.51 cm, *p* < 0.001) between the two different standing postures. Compared with the standard upright position, the natural and comfortable upright position results in a more kyphotic spine profile.

**Conclusion:**

Significant differences in sagittal radiographic parameters were found between the standard upright position and the natural and comfortable upright position; the latter served as a marker for energy conservation during standing and revealed a more kyphotic spinal profile. The standard upright position and natural and comfortable upright position are equally important and should be addressed before a surgical plan is developed for patients who need surgery.

## Background

According to the Scoliosis Research Society (SRS)-Schwab classification, surgery for adult spinal deformities should yield a sagittal vertical axis (SVA) of < 4 cm, a pelvic incidence (PI)-lumbar lordosis discrepancy of < 10° and a pelvic tilt (PT) of < 20° [1] to thereby yield satisfactory patient-reported scores. To determine the need for surgery and deformity correction goals in particular, many studies have used radiographs that were taken after the patient was instructed to stand straight [2], which allows the patient’s capacity to achieve an upright standing posture to be assessed. Several studies have already demonstrated that the degree of improvement in sagittal balance, as assessed by the C7 SVA, is the strongest predictor of improved outcomes in patients with adult spinal deformities [3–6], but Kim et al. suggested that the cranial sagittal vertical axis (CSVA) is a better radiographic measure to predict clinical outcomes of adult spinal deformity surgery than is C7 SVA [7].

After the concept of energy conservation was publicized by Dubousset, according to which an individual can achieve balance with minimal effort [8, 9], research on the upright standing posture in daily life became less important. Optimal total body sagittal alignment (TBSA), from the head to the ankle joint of the human body, may be required to maintain an energy-efficient erect position and a horizontal gaze that is considered clinically satisfactory, and we refer to this posture as the natural and comfortable standing posture. However, few studies have concentrated on this posture, which is commonly assumed in daily life and may explain existing spinal pathologies [10] as well as predict postoperative complications such as proximal junctional failure (PJF) and rod breakages [11–14]. Few studies have reported the differences in sagittal radiographic parameters between the standard upright position and the natural and comfortable upright position. Therefore, we aimed to examine the radiographic differences in between the two different standing postures to obtain additional information beyond what is already known about the standard upright position.

## Methods

Informed consent was obtained from all patients, and the study was approved by the ethics committee of our hospital.

In this study, whole-body radiographs of subjects in both the standard upright position and the natural and comfortable upright position were compared. The inclusion criteria of this study were as follows: 1. an age ranging from 21 to 30 years and 2. a body mass index (BMI) of 18–24. The exclusion criteria of the study were as follows: 1. a history of spine surgery or spinal conditions that do not require surgery; 2. a history of significant back or leg pain (Visual Analogue Scale, VAS score > 3); 3. a personal or family history of a malignancy or significant weight loss within a short period for unexplained reasons; 4. a history of significant trauma to the spine; and 5. the inability to communicate or cooperate properly.

### Radiographic examination and measurements

Based on the inclusion and exclusion criteria listed above, all eligible subjects underwent whole-body radiographs in the standard upright position and the natural and comfortable upright position. All the X-ray images were taken in segments from head to toe and then later reconstructed as one image.

**Fig. 1:**
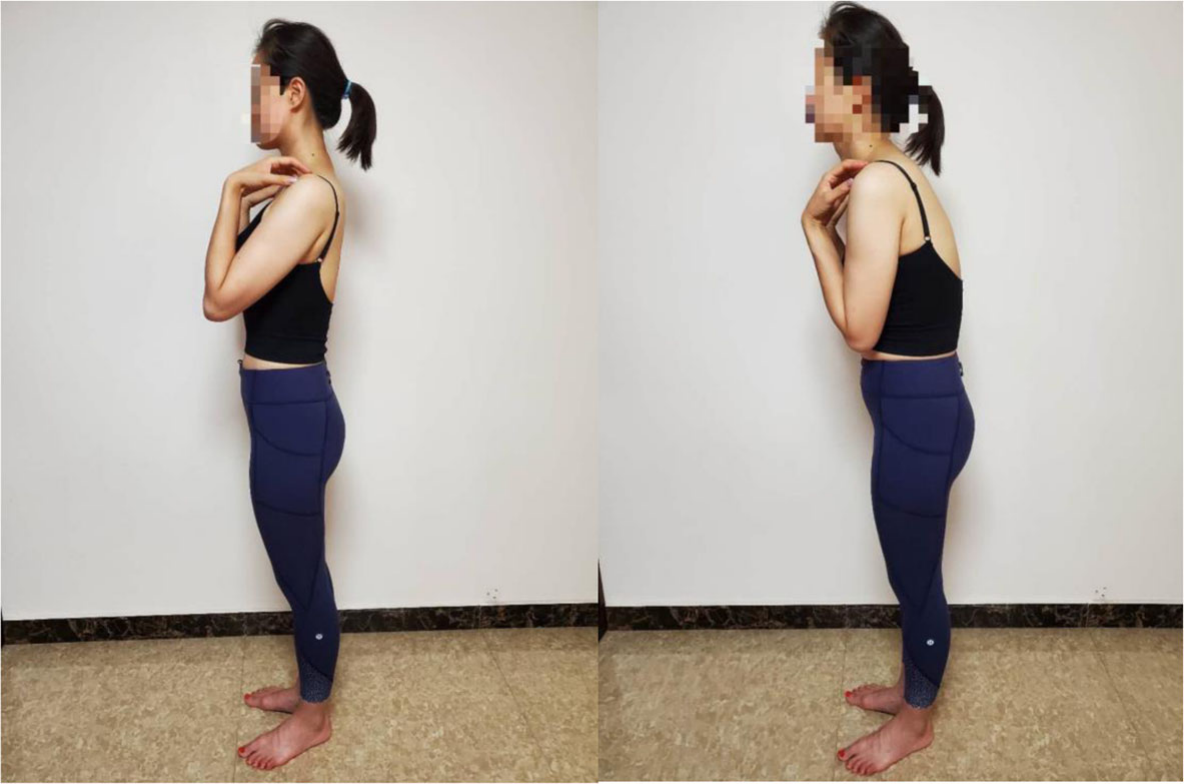
Schematic drawing depicting the landmarks, the parameters and angles used in the measurements: a. the cranial center of mass, b. the posterior tip of the sacrum, c. the center of the femoral heads, d. the center of the knees, e. the center of the ankles ^①^ CSVA-S, ^②^ CSVA-H, ^③^ CSVA-K, ^④^ CSVA-A, ^⑤^ GCA, ^⑥^ GTA, ^⑦^ GLA

Before the X-ray images were taken, the subjects were instructed on how to maintain the standard upright position first through pictorial charts and then the verbal instructions to “stand as straight as possible and do not lean forwards, backwards or to the side, embracing both of the upper limbs in front of the chest”; for the natural and comfortable upright position, all the subjects were told to stand in a way that made him/her feel comfortable and relaxed and then to maintain that posture (Fig. 1).

Radiographic measurements were performed independently by three doctors with more than 2 years of related experience, and the average of the measurements was used for analysis. The parameters assessed included the global cervical angle (GCA, between the inferior end plate of C2 and the inferior end plate of C7), global thoracic angle (GTA, between the superior end plate of T1 and the inferior end plate of T12), and global lumbar angle (GLA, between the superior end plate of L1 and the inferior end plate of L5). These three parameters were used to describe the morphological changes of the cervical spine, thoracic spine and lumbar spine, respectively. The distance from the cranial sagittal vertical axis to the posterior corner of S1 (CSVA-S) and from the cranial sagittal vertical axis to the centers of the hip (CSVA-H), knee (CSVA-K), and ankle (CSVA-A) are thought to be good predictors of clinical outcomes for patients [7].

**Fig. 2:**
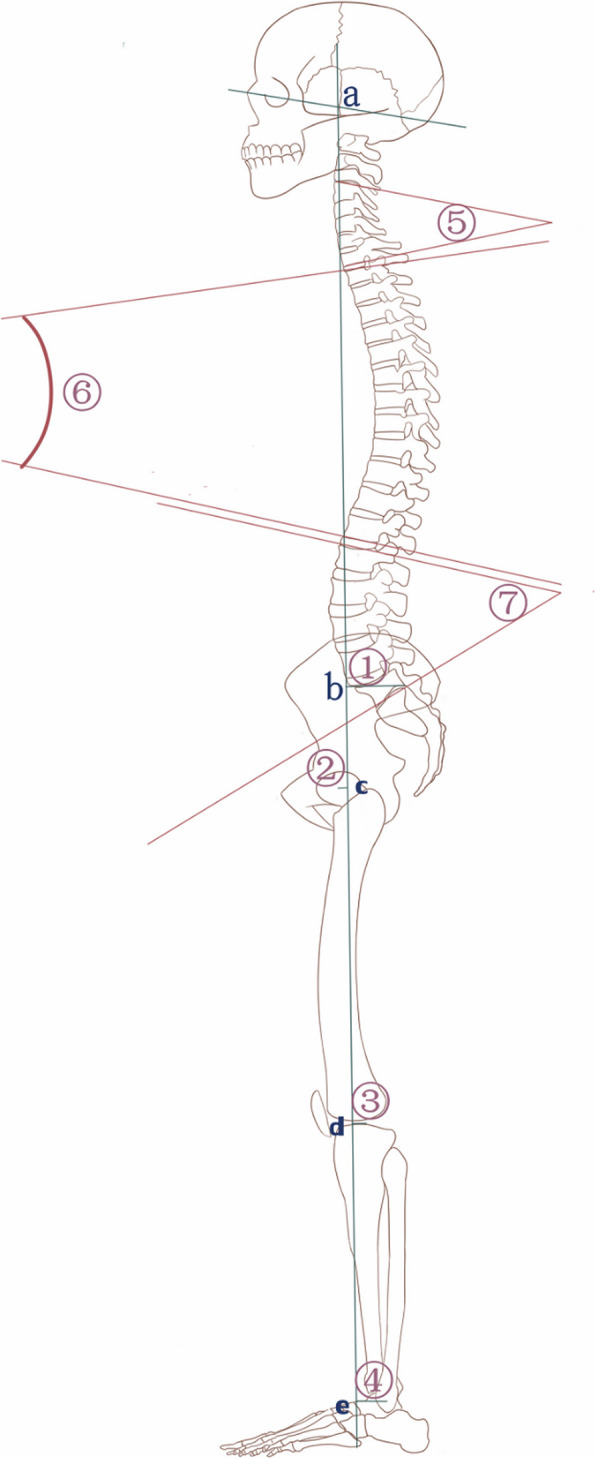
Schematic drawing depicting the landmarks, the parameters and angles used in the measurements: a. the cranial center of mass, b. the posterior tip of the sacrum, c. the center of the femoral heads, d. the center of the knees, e. the center of the ankles, ^①^ CSVA-S, ^②^ CSVA-H, ^③^ CSVA-K, ^④^ CSVA-A, ^⑤^ GCA, ^⑥^ GTA, ^⑦^ GLA

**Fig. 3:**
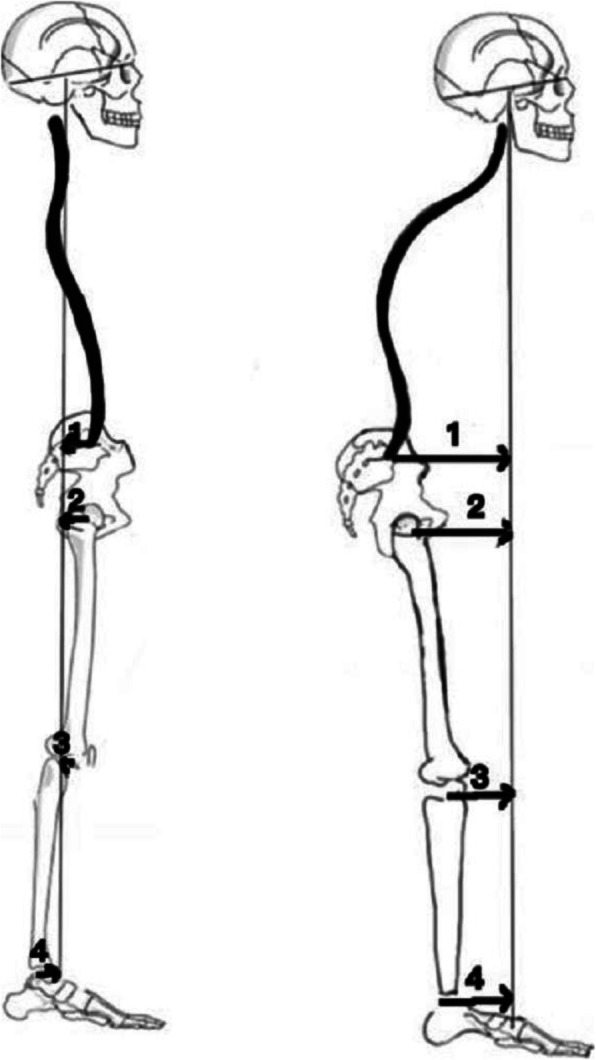
Schematic drawing showing the difference between the two postures about the CSVA parameters

The CSVA parameters were based on the following anatomic landmarks, as shown in Fig. 2: the cranial center of mass (CCM); the posterior, superior corner of the sacrum; and the centers of the hips, knees, and ankles. The CCM was defined as the midpoint of the line connecting the rhinion to the inion. In the lateral view, the center of the hips was defined as the midpoint of the line connecting the centers of the two femoral heads, the center of the knees was the midpoint of the line connecting the centers of the two tibial plateaus, and the center of the ankles was the midpoint of the line connecting the apices of the talar domes [15, 16]. The distance to the sacrum, the hip center, the knee center and the ankle center from the plumb line of the CCM were defined as the CSVA-S, CSVA-H, CSVA-K and CSVA-A, respectively (Fig. 2). If the plumb line of the CCM is in front of the posterior, superior corner of the sacrum, the center of the hip, knees and ankles, the CSVA parameters are counted as positive, and if the plumb line located behind, the CSVA parameters are counted as negative (Fig. 3).

The data were analyzed using Statistical Product and Service Solutions software (version 19.0; SPSS, Chicago, IL). The intraclass correlation coefficient was used to assess the interobserver reliability of the measurements. Paired t tests were used for univariate analysis to compare the radiographic parameters between postures. *P* < 0.05 was considered statistically significant.

## Results

Fifty young and healthy adult subjects (25 males and 25 females) aged 21–30 years were recruited for this study. Multiple significant radiographic differences were found between the standard upright position and the natural and comfortable upright position (Table 1).

**Table 1:** Parameters in the directed and natural standing postures

	Standard upright position (mean ± SD)	Natural and comfortable upright position (mean ± SD)	*P* value
GCA (degree)	17.39 ± 6.90	10.90 ± 3.77	< 0.001
GTA (degree)	25.63 ± 7.27	45.42 ± 8.15	< 0.001
GLA (degree)	42.64 ± 8.05	20.21 ± 7.47	< 0.001
CSVA-S (cm)	0.33 ± 2.76	8.54 ± 3.78	< 0.001
CSVA-H (cm)	1.53 ± 3.11	5.71 ± 3.26	< 0.001
CSVA-K (cm)	3.58 ± 2.47	5.22 ± 2.69	0.002
CSVA-A (cm)	1.79 ± 1.92	4.79 ± 2.51	< 0.001

Compared to the standard upright position, the natural and comfortable upright position showed a more lordotic GCA (10.90 ± 3.77 vs. 17.39 ± 6.90, *p* ≤ .001), a more kyphotic GTA (25.63 ± 7.27 vs. 45.42 ± 8.15 *p* ≤ .001), and a less lordotic GLA (42.64 ± 8.05 vs. 20.21 ± 7.47 *p* ≤ .001). The CSVA measurements were as follows for the standard upright position vs. the natural and comfortable upright position: CSVA-S, 0.33 ± 2.76 cm vs. 8.54 ± 3.78 cm, *p* < 0.001; CSVA-H, 1.53 ± 3.11 cm vs. 5.71 ± 3.26 cm, *p* < 0.001; CSVA-K, 3.58 ± 2.47 cm vs. 5.22 ± 2.69 cm, *p* = 0.002; and CSVA-A, 1.79 ± 1.92 cm vs. 4.79 ± 2.51 cm, *p* < 0.001 (Table 1). The interobserver reliability of the angle measurements was very good (K = 0.863).

## Discussion

The spine provides structural support for the body and transfers the weight of the upper body to the lower extremities via the pelvis. To maintain whole-body balance, a balance between lordosis and kyphosis is needed; then, a horizontal gaze can be achieved [17]. The restoration of lordosis in the lower lumbar segments may be an appropriate goal for spinal realignment surgeries, as previous studies have shown that undercorrection is associated with a low proximal junctional kyphosis (PJK) rate [18, 19]. Our findings regarding the natural and comfortable standing position may be helpful in understanding the effects of lordotic undercorrection, as well as the potential value of restoring lordosis in the lower lumbar spine to reduce biomechanical complications. However, some postoperative complications, such as PJK/PJF [11–13] and rod breakage [14], still remain biomechanical issues of unknown causes following surgery. When the postoperative results were evaluated with respect to the radiographic parameters within the spinal-pelvic area [3–6, 20, 21], such as C7 SVA and PT, the total body sagittal alignment from the skull to the ankle joint was ignored, which may influence the patient-reported outcomes.

In our clinic, some patients showed relatively poor improvement in clinical scores, although we used C7 SVA to assess the improvement in spinal sagittal balance after surgical correction. The C7 SVA, which is defined as the plumb line from the 7th cervical vertebra to the sacrum, can only be used in the evaluation of thoracic and lumbar spine; it cannot be used in the evaluation of the whole spine, cervical spine, or lower limbs, so it is not sufficient for evaluating the global balance of a patient [22–24]. Not only the spine itself but also the pelvis and lower limbs are involved in compensatory strategies when spinal imbalance occurs. The spinopelvic movement at the hip joint involves rotational actions about the hip center, which are determined by both pelvic retroversion and backward femoral inclination. Knee flexion as well as ankle flexion follows to achieve full-body sagittal balance after maximum hip compensation is achieved. If the spine, hip joints, knee joints, and ankle joints are considered a linear chain, the knee joints are the most active parts of the chain, in addition to the spine. We speculated that the reason for this finding is that the hip joints are fixed in the pelvis and that the movement of the ankle joints is restricted by the ground.

Kim et al. [7] suggested that the distance from the cranial sagittal vertical axis to the ankle joint (CrSVA-A) is a radiographic parameter that can predict the widest range of patient-reported outcomes, whereas C7 SVA is significantly associated with the ODI and only three of the SRS subscores (pain, function, and total score). The CrSVA-A (Global SVA), linking the head to the ankle joint, showed a strong correlation with the SRS satisfaction subscore in a retrospective radiographic and clinical analysis in 108 ASD patients. Hey et al. [18] considered that the changes that occur with age are likely induced by relaxed postural tendencies. Based on these conclusions, we think that using only radiographic parameters from part of the body might not be sufficient to fully determine the clinical outcomes; thus, in predicting the postoperative efficacy of treatments in adult patients with spinal deformities, spinal-pelvic factors alone are not sufficient, and parameters of the head and lower limbs should also be considered. The occurrence of PJK/PJF and rod failure in both old and young individuals evoke a mechanical problem caused by the standing posture that may increase the risk of mechanical complications. In ASD surgery, due to the evolved physiology of older individuals and the weaker muscles and ligaments, elderly individuals may be at high risk of complications, so the surgical strategy was improved to minimize age-related mechanical complications such as PJK/PJF; however, such complications can occur in younger patients following deformity correction, which indicates that a mechanical problem that affects both old and young patients persists. Different standing postures yielded different spinal profiles and affected the angle of the pedicle screws, the arc of the connecting rods and the shear force of the entire implant system. Although we do not clearly understand the impact of the standing posture on postoperative complications, we believe that in the process of deformity correction, a natural and comfortable upright position should be given equal attention as the standard upright position.

Although the findings of this current study in young, healthy adults cannot be directly generalized to ASD patients until the reproducibility of these concepts in ASD patients is assessed in another study, we noticed that when a subject changes from the standard upright position to the natural and comfortable standing position, the overall degree of kyphosis in the spine becomes larger, consistent with the changes that occur due to aging.

This study still has limitations. First, the limited ethnic backgrounds of the subjects can be considered a limitation. Second, pelvic morphology is known differ between sexes; taking the strong relationship between lumbar morphology and pelvic morphology into account, potential bias due to pelvic morphological effects between sexes should be considered.

## Conclusion

We believe that the standard upright position and natural and comfortable upright position are equally important and should be addressed before a surgical plan is developed for patients who need surgery.

## Data Availability

The datasets used or analyzed during the study are available from the corresponding author on reasonable request.

## Abbreviations

GCA: Global Cervical Angle
GTA: Global Thoracic Angle
GLA: Global Lumbar Angle
CSVA: Cranial Sagittal Vertical Axis
CSVA-S: Cranial Sagittal Vertical Axis to the posterior corner of S1
CSVA-H: Cranial Sagittal Vertical Axis to the center of the hip
CSVA-K: Cranial Sagittal Vertical Axis to the center of the knee
CSVA-A: Cranial Sagittal Vertical Axis to the ankle
SRS: Scoliosis Research Society
PI: Pelvic incidence
SVA: Sagittal Vertical Axis
PT: Pelvic Tilt
PJF: Proximal Junctional Failure
BMI: Body Mass Index
VAS: Visual Analog Scale
CCM: Cranial Center of Mass
ODI: Oswestry Disability Index
ASD: Adult spinal deformity
